# Predictive analytics and tailored interventions improve clinical outcomes in older adults: a randomized controlled trial

**DOI:** 10.1038/s41746-021-00463-y

**Published:** 2021-06-10

**Authors:** Sara Bersche Golas, Mariana Nikolova-Simons, Ramya Palacholla, Jorn op den Buijs, Gary Garberg, Allison Orenstein, Joseph Kvedar

**Affiliations:** 1 Partners Connected Health Innovation, Partners HealthCare, Boston, MA USA; 2 Philips Research, Eindhoven, The Netherlands; 3 Massachusetts General Hospital, Boston, MA USA; 4 Harvard Medical School, Boston, MA USA; 5 Tufts University School of Medicine, Department of Public Health and Community Medicine, Boston, MA USA; 6 Partners HealthCare at Home, Waltham, MA USA; 7 Philips Population Health Management, Framingham, MA USA

**Keywords:** Randomized controlled trials, Outcomes research, Population screening

## Abstract

This study explored the potential to improve clinical outcomes in patients at risk of moving to the top segment of the cost acuity pyramid. This randomized controlled trial evaluated the impact of a Stepped-Care approach (predictive analytics + tailored nurse-driven interventions) on healthcare utilization among 370 older adult patients enrolled in a homecare management program and using a Personal Emergency Response System. The Control group (CG) received care as usual, while the Intervention group (IG) received Stepped-Care during a 180-day intervention period. The primary outcome, decrease in emergency encounters, was not statistically significant (15%, *p* = 0.291). However, compared to the CG, the IG had significant reductions in total 90-day readmissions (68%, *p* = 0.007), patients with 90-day readmissions (76%, *p* = 0.011), total 180-day readmissions (53%, *p* = 0.020), and EMS encounters (49%, *p* = 0.006). Predictive analytics combined with tailored interventions could potentially improve clinical outcomes in older adults, supporting population health management in home or community settings.

## Introduction

Worldwide, older adults are becoming the largest population due to increasing longevity and lower fertility rates^1^. In the United States, it is estimated that by 2050, the proportion of adults aged 65 years and older will nearly double^2^. Multiple chronic conditions are prevalent in this population and are the most common cause of emergency department (ED) and hospital (re)admissions^3,4^. Further, management of these chronic conditions in acute care settings contribute to more than two-thirds of total healthcare costs^5,6^. These increasing costs necessitate the development of innovative digital health strategies to enable healthcare providers to achieve better health outcomes at lower costs. This marks a fundamental shift towards outcomes-based care and leverages proactive population health management to achieve the quadruple aim—improved health outcomes, patient and clinician satisfaction, and reduced costs. In addition, the COVID-19 pandemic profoundly emphasizes the need for shifting from reactive care at the hospital to proactive care at home^7^. This proactive approach can have a significant impact on the most vulnerable patients—older adults with comorbidities.

Partners HealthCare at Home (PHH) is a preventative homecare management system that offers general care as well as specialized services to patients within the Mass General Brigham (MGB). MGB is an integrated delivery network located in Massachusetts, comprised of two academic medical centers as well as community hospitals. The PHH population health management program leverages connected health technologies, including a Personal Emergency Response System (PERS).

PERS helps older adults to live independently at home by providing access to immediate assistance in case of medical emergencies that could lead to costly ED visits and hospitalizations. PERS is a remote patient monitoring service consisting of three components: (1) a help push button that is worn as a necklace or on the wrist, (2) an in-home communication system and (3) an emergency 24/7 response center. Patients can press the “help” button at any time to activate the in-home communication system that connects to the response center. The response center associate obtains information about the situation and contacts either an informal responder (e.g., neighbor, a family member) or an Emergency Medical Service (EMS, e.g., ambulance, police, or fire department) based on the patient’s specific situation, and then follows up to confirm that help has arrived. We used PERS data of more than 500,000 users to develop and validate a predictive model that predicts the risk of 30-day ED transport use^8^. This algorithm is the foundation of CareSage, a care provider-facing patient risk assessment system that supports healthcare providers in assessing patient’s risk of ED transport so they can proactively intervene and potentially prevent unnecessary healthcare utilization in older patients.

Healthcare expenditures in the United States are unevenly distributed across individuals and different segments of the population^9,10^. For example, the bottom 50% least expensive patients (B segment) spend only 3–4% on healthcare, whereas the top 5% most expensive patients (T segment) spend 50% of the total expenditures. The middle 45% of the population (M segment) accounts for the remaining 45% of the total cost. Accordingly, most healthcare organizations (HCOs) develop care management programs targeting the T segment^11,12^. However, our longitudinal retrospective study of healthcare costs of an older population has shown that the M segment was persistently the costliest through all 5 years with the highest expected annual cost increase compared with the other segments^6^. These findings highlight why this study focused on patients in the M segment, which, by definition, has nine times more patients than the T segment, and why we used predictive analytics to specifically identify M segment patients at high risk of adverse events. This risk identification is the first step in the Stepped-Care approach used as the intervention in this study, which triggers nurse-driven care plans tailored to risk-flagged patients as the second step of the intervention.

The study objective was to evaluate the impact of this Stepped-Care approach on the healthcare utilization and healthcare costs of older patients using PERS.

## Results

### Participants flow

**Fig. 1:**
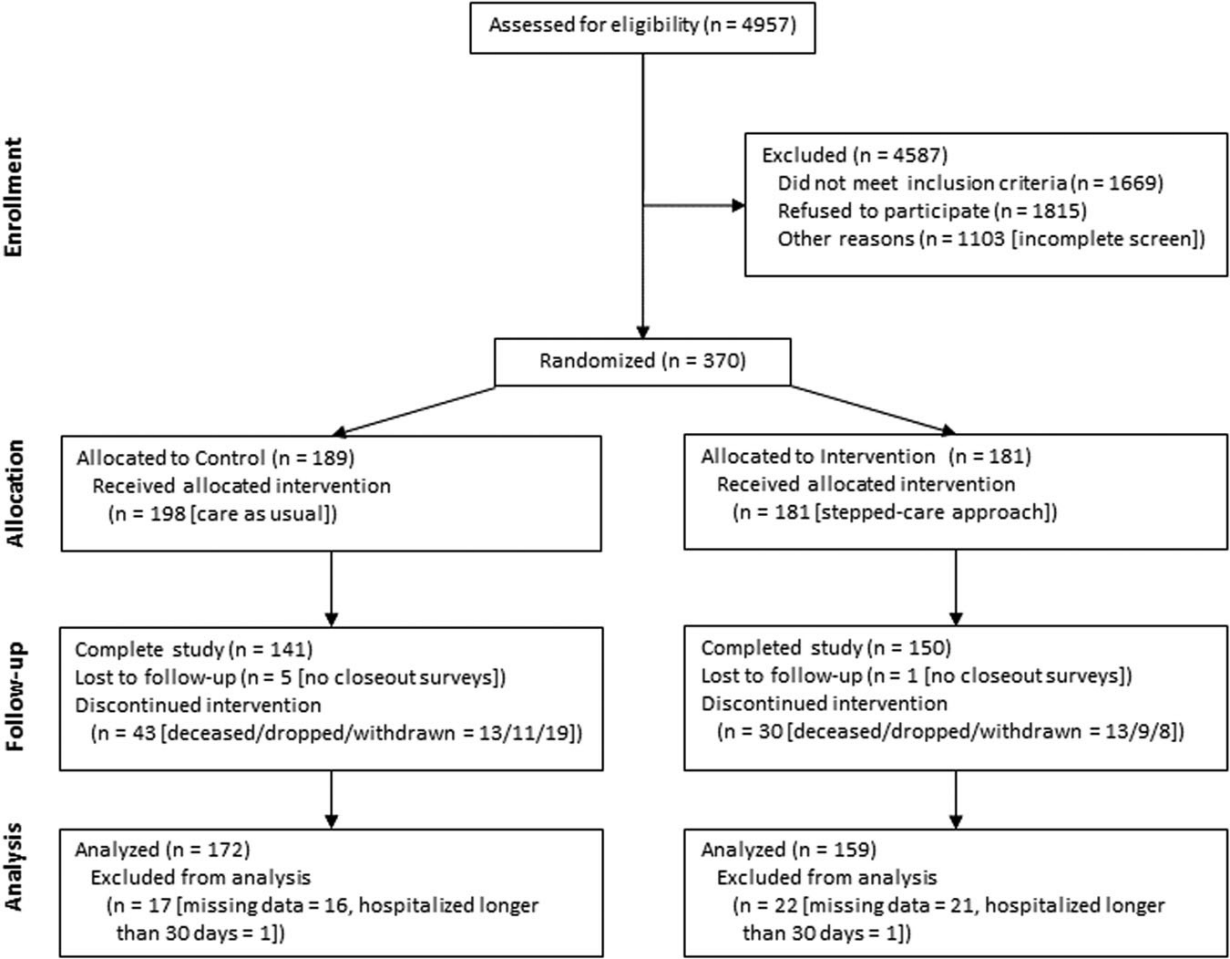
Participant flowchart. Figure 1 summarizes the recruitment, randomization, and retention flow of patients in this study, leading to the final analyzed cohort.

The participant flow through the study is illustrated in Fig. 1. A total of 4957 patients were assessed for potential eligibility in the study. Of these 4957 patients, 1669 (34%) were ineligible, 1815 (37%) were disinterested in the study, and 1103 (22%) could not be screened (were deceased or unavailable). Enrollment began in May 2017 and ended in July 2018 after enrolling 370 patients. Patients were randomly assigned to either the CG (*n* = 189) or IG (*n* = 181). After excluding the patients with missing data (16 in the CG and 21 in the IG) and those hospitalized longer than 30 days (1 in each group), there were 172 (91%) and 159 (88%) patients in the CG and IG (total *n* = 331), respectively, included in the intention-to-treat analysis. Patients who died, were dropped, withdrawn, or lost to follow-up were included in the data analysis with incomplete data, i.e., data only for the period they participated in the study. The final patient closed-out in April 2019.

### Baseline characteristics

There were no statistically significant differences between the baseline characteristics of the CG and IG (see Tables 1 and 2). The overall study population (*n* = 331) had a median age of 80 years and were primarily female (67%), white (85%), widowed (44%), with a college degree or higher (46%), and retired (87%) (Table 1). The most common comorbidities were hypertension (60%), inflammatory pain disorders (such as arthritis, fibromyalgia, etc., 58%), high cholesterol (36%), and cancer (31%). Nearly two-thirds (61%) of patients reported having at least 3 pre-existing conditions (Table 2).

**Table 1:** Baseline characteristics by group—demographics.

Baseline characteristics	Population *n* = 331	Control *n* = 172	Intervention *n* = 159	*p*-value
**Study status,** ***n*** **(%)**				0.198
Closed out	258 (77.9)	129 (75.0)	129 (81.1)	
Withdrawn	27 (8.2)	19 (11.0)	8 (5.0)	
Deceased	26 (7.9)	13 (7.6)	13 (8.2)	
Dropped	20 (6.0)	11 (6.4)	9 (5.7)	
**Existing lifeline subscribers,** ***n*** **(%)**	112 (33.6)	53 (30.6)	59 (36.9)	0.277
**Gender, Male,** ***n*** **(%)**	108 (32.6)	57 (33.1)	51 (32.1)	0.929
**Age, median years (IQR)**	80 (74, 86)	80 (74, 86)	81 (74, 87)	0.441
**Race,** ***n*** **(%)**				0.601
White	282 (85.2)	142 (82.6)	140 (88.1)	
Black or African American	27 (8.2)	18 (10.5)	9 (5.7)	
Asian	7 (2.1)	4 (2.3)	3 (1.9)	
Hispanic or Latino (any race)	6 (1.8)	4 (2.3)	2 (1.3)	
Other or more than one race	6 (1.8)	3 (1.7)	3 (1.9)	
Unknown	3 (0.9)	1 (0.6)	2 (1.3)	
**Marital status,** ***n*** **(%)**				0.239
Widowed	146 (44.1)	79 (45.9)	67 (42.1)	
Married or partnered	101 (30.5)	47 (27.3)	54 (34.0)	
Divorced or separated	45 (13.6)	26 (15.1)	19 (11.9)	
Single, never been married	36 (10.9)	20 (11.6)	16 (10.1)	
Other or unknown	3 (0.9)	0 (0.0)	3 (1.9)	
**Living with someone (vs. alone),** ***n*** **(%)**	168 (50.8)	80 (46.5)	88 (55.3)	0.135
**Educational level,** ***n*** **(%)**				0.424
Less than high school	22 (6.6)	13 (7.6)	9 (5.7)	
High school or GED	84 (25.4)	45 (26.2)	39 (24.5)	
Some college or vocational/technical training	71 (21.5)	42 (24.4)	29 (18.2)	
College graduate	69 (20.8)	31 (18.0)	38 (23.9)	
Post-graduate degree	84 (25.4)	40 (23.3)	44 (27.7)	
Other or unknown	1 (0.3)	1 (0.6)	0 (0.0)	
**Employment status,** ***n*** **(%)**				0.664
Retired	288 (87.0)	154 (89.5)	134 (84.3)	
Disabled	15 (4.5)	5 (2.9)	10 (6.3)	
Employed	14 (4.2)	6 (3.5)	8 (5.0)	
Homemaker	5 (1.5)	3 (1.7)	2 (1.3)	
Unemployed	4 (1.2)	2 (1.2)	2 (1.3)	
Other or unknown	5 (1.5)	2 (1.2)	3 (1.9)

**Table 2:** Baseline characteristics by group—comorbidities.

Baseline characteristics	Population *n* = 331	Control *n* = 172	Intervention *n* = 159	*p*-value
**Comorbidities,** ***n (%)***				
Hypertension	199 (60.1)	104 (60.5)	95 (59.7)	0.983
Inflammatory pain disorders	191 (57.7)	101 (58.7)	90 (56.6)	0.781
High cholesterol	120 (36.3)	55 (32.0)	65 (40.9)	0.117
Cancer	101 (30.5)	54 (31.4)	47 (29.6)	0.808
Chronic heart disease	72 (21.8)	37 (21.5)	35 (22.0)	1.000
Diabetes	66 (19.9)	40 (23.3)	26 (16.4)	0.152
Depression	60 (18.1)	31 (18.0)	29 (18.2)	1.000
Chronic obstructive pulmonary disorder	53 (16.0)	30 (17.4)	23 (14.5)	0.557
Asthma	49 (14.8)	25 (14.5)	24 (15.1)	1.000
Stroke	50 (15.1)	29 (16.9)	21 (13.2)	0.439
Congestive heart failure	43 (13.0)	24 (14.0)	19 (11.9)	0.705
Acute myocardial infarction	43 (13.0)	27 (15.7)	16 (10.1)	0.174
Other	45 (13.6)	20 (11.6)	25 (15.7)	0.355
None	13 (3.9)	7 (4.1)	6 (3.8)	1.000
**Total number of comorbidities,** ***n*** **(%)**				0.867
0	13 (3.9)	7 (4.1)	6 (3.8)	
1	43 (13.0)	22 (12.8)	21 (13.2)	
2	72 (21.8)	36 (20.9)	36 (22.6)	
3	77 (23.3)	37 (21.5)	40 (25.2)	
≥4	126 (38.1)	70 (40.7)	56 (35.2)	

We adjusted for these patient characteristics in the regression models used to analyze healthcare outcomes, then conducted analysis of variance (ANOVA) to compare adjusted and unadjusted models. Because none of the adjusted models crossed the *p* < 0.05 threshold, we selected the parsimonious unadjusted models to present in the healthcare outcomes results section below.

### Stepped-Care approach

The first step of this approach is the generation of risk scores by the predictive model. While only the risk-scores of the IG patients were shown to the nurse on the CareSage dashboard, at the backend the predictive model used the PERS utilization data of patients in both groups to calculate their risk scores daily. These detailed data were extracted and analyzed at the end of the study period. Similar proportions of patients in both groups were ever flagged as high risk according to the model: *n* = 42 (24.4%) in the CG and *n* = 39 (24.5%) in the IG; *p* = 1.00. These results suggest no significant latent between-groups difference in patients at high-risk. The second step of the Stepped-Care approach is study nurse triage of high-risk patients, which included a needs assessment questionnaire and recommendations for further care tailored to the patient’s needs.

Table 3 summarizes the number of patients who received each step of the Stepped-Care approach, as well as the types of tailored recommendations, their frequency and proportion of patients in the IG receiving them. Out of the 39 IG patients flagged by the model as high-risk, 34 received an initial nurse needs assessment and feedback (5 of the 39 patients who were flagged as high-risk were unreachable for assessment at the time). Beyond the initial assessment and feedback, 32 patients (out of 34) were reassessed and received additional feedback multiple times during their intervention period, resulting in 127 follow-up reassessments and feedbacks. Additional follow-up components were phone education, nurse home visits, physical therapy/exercise, PCP referral, home health aide recommendation, appointment adherence, medication regimen advice, mental health services, and transportation assistance. Each of these interventions was received by at most 5 patients.

**Table 3:** Patients in the IG receiving each step of the Stepped-Care approach and types of tailored interventions.

**Step 1: Predictive model risk assessment**	**Patients**	**Assessments**
**Patients with risk assessment,** ***N***	159	
Patients flagged as high risk, *n* (% of *N*)	39 (25)	
**Step 2: Nurse-driven tailored interventions**		
**High risk patients,** ***N***	39	
Patients assessed, *n* (% of *N*)	34 (87)	
Assessments per patient:		
Range	1–6	
Median (IQR)	6 (3,6)	
**Total assessed patients/assessments,** ***N***	34	161*
Assessments resulting in follow-up interventions, *n* (% of *N*)	11 (32)	18 (11)
Types of follow-up interventions, *n* (% of *N*)		
Phone education	5 (15)	5 (3)
Nurse home visits	4 (12)	5 (3)
Physical therapy/exercise	4 (12)	5 (3)
Referral to PCP	3 (9)	5 (3)
Home Health Aide	3 (9)	3 (2)
Appointment adherence	2 (6)	2 (1)
Others (e.g., medication regimen, mental health service, transportation)	3 (9)	3 (2)

*161 total assessments constitute of 34 initial assessments and 127 reassessments.

### Healthcare outcomes

Table 4 summarizes the adverse events in the IG and CG for both “intention-to-treat” and “per-protocol” approaches. Table 5 provides the results of the inferential analysis of the outcome rates. The regression model coefficients with their 95%CI, the associated *p*-values and the adjusted levels of significance using Benjamini & Hochberg (BH) correction are reported. A comparison of the two approaches is provided in the Discussion section and Table 6, however, for the sake of conciseness, the paragraphs below focus on the results of the more conservative intention-to-treat analysis rather than the per-protocol analysis.

**Table 4:** Summary of healthcare outcomes.

		Intention-to-treat		Per-protocol	
		CG, *n* = 172	IG, *n* = 159*	CG, *n* = 129	IG, *n* = 129
**ED encounters**	Total events, *n*	99	78	76	61
	Patients with event, *n* (%)	55 (32.0)	48 (30.2)	41 (31.8)	40 (31.0)
**Hospital admissions**	Total events, *n*	58	46	44	35
	Patients with event, *n* (%)	37 (21.5)	37 (23.3)	27 (20.9)	30 (23.3)
**30-day readmissions**	Total events, *n*	15	6	11	4
	Patients with event, *n* (%)	12 (7.0)	4 (2.5)	8 (6.2)	2 (1.6)
**90-day readmissions**	Total events, *n*	24	7	20	4
	Patients with event, *n* (%)	17 (9.9)	4 (2.5)	13 (10.1)	2 (1.6)
**180-day readmissions**	Total events, *n*	32	14	27	9
	Patients with event, *n* (%)	18 (10.5)	11 (6.9)	14 (10.9)	7 (5.4)
**EMS encounters**	Total events, *n*	53	25	49	23
	Patients with event, *n* (%)	29 (16.9)	20 (12.7)	26 (20.2)	18 (14.0)
**ED transports**	Total events, *n*	34	21	33	20
	Patients with event, *n* (%)	21 (12.2)	16 (10.1)	20 (15.5)	15 (11.6)

*For PERS outcomes (EMS encounters and ED transport), an outlier with extremely high PERS utilization was excluded in the Intention-to-treat analysis (*n* = 158).

**Table 5:** Inferential analysis of healthcare outcomes rates.

Healthcare outcomes rates	Intention-to-treat				
	CG, *n* = 172	IG, *n* = 159*	Model coef. (95% CI)	*p*-value**	*α*_adj_ ***
**ED encounters rate**, mean (sd)	0.58 (1.12)	0.49 (0.94)	0.852 (0.632, 1.145)	0.291	0.05 (0.05*7/7)
**Hospital admissions rate**, mean (sd)	0.34 (0.75)	0.29 (0.61)	0.858 (0.580, 1.261)	0.438	0.0429 (0.05*6/7)
**30-day readmissions rate**, mean (sd)	0.09 (0.36)	0.04 (0.27)	0.433 (0.154, 1.064)	0.083	0.0286 (0.05*4/7)
**90-day readmissions rate**, mean (sd)	0.14 (0.50)	0.04 (0.30)	0.316 (0.126, 0.695)	0.007	0.0143 (0.05*2/7)
**180-day readmissions rate**, mean (sd)	0.19 (0.62)	0.09 (0.36)	0.472 (0.248, 0.869)	0.020	0.0214 (0.05*3/7)
**EMS encounters rate**, mean (sd)	0.31 (0.95)	0.16 (0.46)	0.513 (0.314, 0.817)	0.006	0.00714 (0.05*1/7)
**ED transports rate**, mean (sd)	0.20 (0.64)	0.13 (0.44)	0.672 (0.384, 1.149)	0.153	0.0357 (0.05*5/7)

*For PERS outcomes (EMS encounters and ED transports), an outlier with extremely high PERS utilization was excluded in the Intention-to-treat analysis; **p-values of Poisson regression models; ***α_adj_ is the statistical significant threshold *α*=0.05 adjusted for multiple testing by Benjamini–Hochberg correction, i.e., *α*_adj_ = α*i/7, where *i* = 1…7 is the position or the pi-value in an ordered list from smallest to largest.

**Table 6:** Comparison of study outcomes from intention-to-treat and per-protocol analyses.

Outcomes rate (IG < CG)	Intention-to-treat			Per-protocol			Multiple testing correction - BH
Statistically significant	Yes *p* ≤ *α*_adj_	Close to *α*_adj_ < *p* ≤ 0.1	No *p* > 0.1	Yes *p* ≤ α_adj_	Close to *α*_adj_ < *p* ≤ 0.1	No *p* > 0.1	α_adj_*
ED encounters			0.291			0.201	0.0500
Hospital admissions			0.438			0.312	0.0429
30-day readmission		0.083			0.082		0.0286
90-day readmission	0.007			0.003			0.0143
180-day readmission	0.020			0.004			0.0214
EMS encounters	0.006			0.003			0.0071
ED transport			0.153		0.077		0.0357

*α_adj_ is the statistically significant threshold *α*=0.05 adjusted for multiple testing by BH correction.

ED Encounters: There were 55 patients (32.0%) having a total of 99 ED encounters in the CG versus 48 patients (30.2%) with a total of 78 ED encounters in the IG (Table 4, intention-to-treat). The ED encounters rate in the IG was 15% lower than the CG (regression model coefficient 0.85, 95% CI = 0.63–1.15), however, it was not statistically significant (*p* = 0.291), see Table 5.

Hospital admissions: There were 37 patients (21.5%) having a total 58 hospital admissions in the CG versus 37 patients (23.3%) with a total of 46 hospital admissions in the IG (Table 4, intention-to-treat). The hospital admissions rate in the IG was 14% lower than the CG (regression model coefficient 0.86, 95% CI = 0.58–1.26), however it was not statistically significant (*p* = 0.438), see Table 5.

30-day readmissions: There were 12 patients (7.0%) having a total of 15 hospital 30-day readmissions in the CG versus 4 patients (2.5%) with a total of 6 hospital 30-day readmissions in the IG (Table 4, intention-to-treat). The 30-day readmissions rate in the IG was 57% lower than the CG (regression model coefficient 0.43, 95% CI = 0.15–1.06), but was not statistically significant (*p* = 0.083), see Table 5.

90-day readmissions: There were 17 patients (9.9%) having a total of 24 hospital 90-day readmissions in the CG versus 4 patients (2.5%) with a total of 7 hospital 90-day readmissions in the IG (Table 4, intention-to-treat). The 90-day readmissions rate in the IG was 68% lower than the CG (regression model coefficient 0.32, 95% CI = 0.13–0.70) and it was statistically significant (*p* = 0.007), see Table 5. Also, the number of patients having 90-day readmissions in the IG was 76% lower than the CG (regression model coefficient 0.24, 95% CI = 0.07–0.65) and it was statistically significant (*p* = 0.011).

180-day readmissions: There were 18 patients (10.5%) having a total of 32 hospital 180-day readmissions in the CG versus 11 patients (6.9%) with a total of 14 hospital 180-day readmissions in the IG (Table 4, intention-to-treat). The 180-day readmissions rate in the IG was 53% lower than the CG (regression model coefficient 0.47, 95% CI = 0.25–0.87) and it was statistically significant (*p* = 0.020), see Table 5.

EMS encounters: There were 29 patients (16.9%) having a total of 53 EMS encounters in the CG versus 20 patients (12.7%) with a total of 25 EMS encounters in the IG (Table 4, intention-to-treat). The EMS encounters rate in the IG was 49% lower than the CG (regression model coefficient 0.51, 95% CI = 0.31–0.82) and it was statistically significant (*p* = 0.006), see Table 5.

ED transports: There were 21 patients (12.2%) having a total of 34 ED transports in the CG versus 16 patients (10.1%) with a total of 21 ED transports in the IG (Table 4, intention-to-treat). The ED transports rate in the IG was 33% lower than the CG (regression model coefficient 0.67, 95% CI = 0.38–1.15), however it was not statistically significant (*p* = 0.153), see Table 5.

## Discussion

The analyses revealed some key findings in comparing the healthcare utilization of both groups. While the study was powered to detect a 35% decrease in ED encounters rate between CG and IG, the results demonstrated a 15% decrease, not statistically significant at *p* = 0.291. However, the IG had 68% fewer 90-day readmissions (*p* = 0.007) compared to the CG with a corresponding 76% decrease of patients with any 90-day readmissions (9.9% control vs. 2.5% intervention group, *p* = 0.011), as well as 53% fewer 180-day readmissions (*p* = 0.020) compared to the CG. EMS utilization went also down, with the IG having 49% fewer EMS encounters (*p* = 0.006) compared to the CG. Other outcomes that decreased in the IG compared to the CG but did not reach statistical significance include hospital admissions (14% decrease, *p* = 0.438), 30-day readmission rates (57% decrease, *p* = 0.083), and ED transports (33% decrease, *p* = 0.153).

One of the reasons for non-significant findings with respect to the primary and some of the secondary outcomes might be the nature of the study design: only patients predicted at high-risk during Step 1 received Step 2 of the study intervention, which was only 34 (21%) out of 159 IG patients. This, in addition to inherent variation in the implementation of Step 2 due to tailoring (potentially creating high within-group variability), may have diluted the intervention effect and/or reduced the power to detect significant intervention effects. Additionally, patients might have EMS encounters and ED transports initiated outside of the PERS service by calling 911 or being referred by their PCP. Since both adverse events in the patient’s history are important predictors of upcoming ED encounters the performance of the model predicting high risk patients in the IG might have be affected as well.

The results aforementioned are based on the intention-to-treat approach which is known to under-estimate the intervention effects compared to the per-protocol approach^13^. However, the latter is biased to patients who completed the intervention. Table 6 summarizes the similarities and differences between both approaches. First: 90-day readmissions, 180-day readmissions, and EMS encounters were statistically significant in both, with a stronger *p*-value in the per-protocol analysis compared to the intention-to-treat analysis. Second: 30-day readmissions, ED encounter, and hospital admissions were not statistically significant in either analysis, although 30-day readmissions approached significance. Finally, ER transports had a stronger *p*-value in the per-protocol analysis relative to the intention-to-treat analysis, though only approached significance.

Our previous study^14^ showed that 30-day readmission rates over a 5-year period were relatively steady and lower than the national level whereas 90- and 180-day readmissions increased significantly, along with the number of overall admissions. These findings reiterate those from a previous study which showed that 30-day readmission rates at the national level fell sharply as a result of the Accountable Care Act^15^. However, evidence from prior research is inconclusive regarding how 90- and 180-day readmission rates were affected by innovative population management strategies implemented by HCOs to reduce readmissions^16^. Thus, this study provides important evidence that a population management strategy based on a Stepped-Care approach that combines a predictive algorithm with a nurse-driven tailored intervention could potentially drive down 90- and 180-day readmission rates in older patients.

It is also important to note that the tailored intervention in this study relied on nurse expertise to effectively deliver it and there was no intervention protocol designed by the study that could be easily reproduced. Therefore, applying the same Stepped-Care approach to a healthcare system outside MGB might be difficult. This issue of limited external validity is a known weakness in hospital readmissions reduction programs^15,16^.

**Fig. 2:**
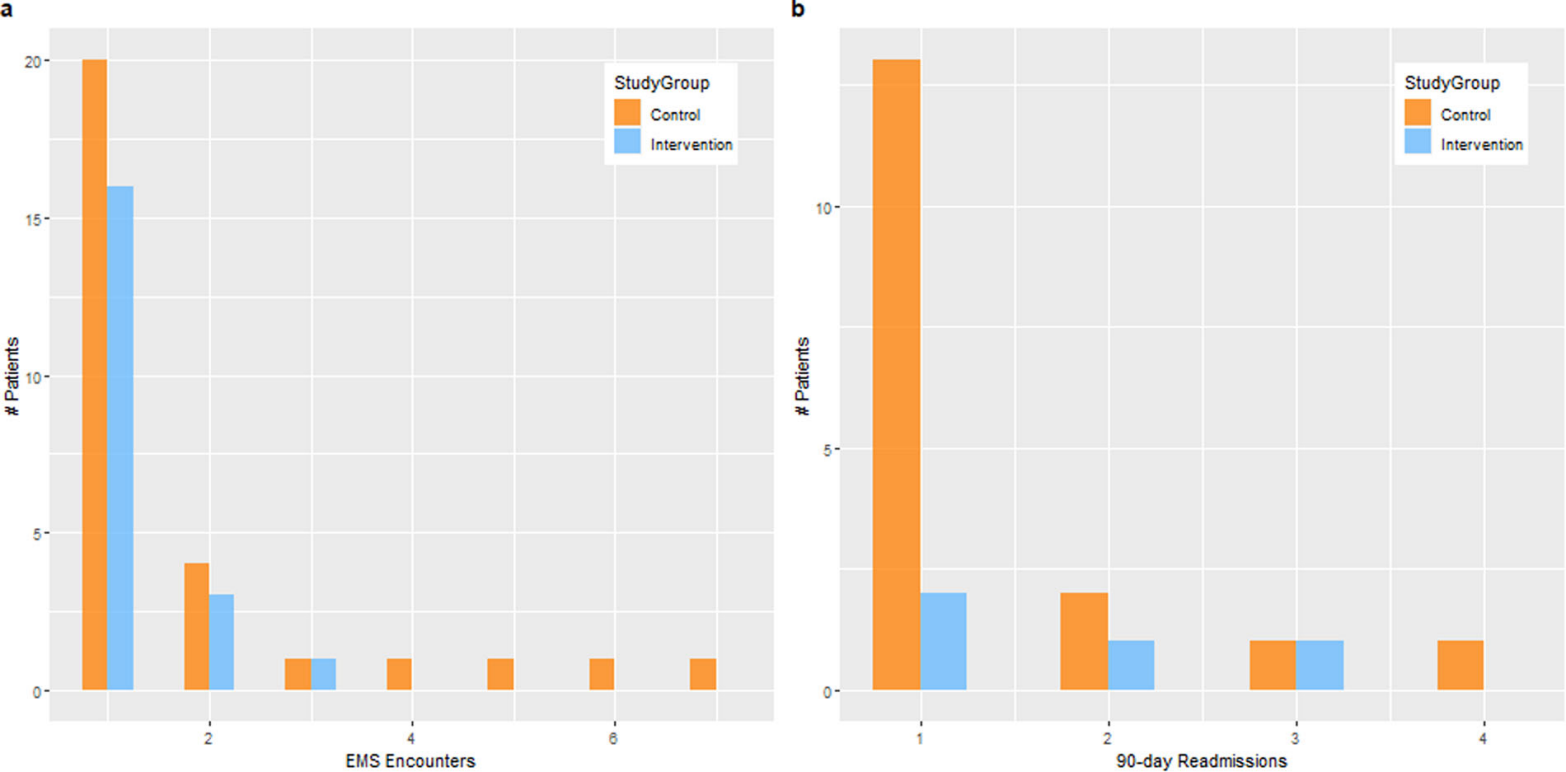
Patients with single vs. multiple (a) EMS encounters and (b) 90-day readmissions. Figure 2 summarizes between-groups differences in patients with 1 event versus those with multiple events for EMS encounter and 90-day readmission events.

The statistically significant decrease of event rates (90- and 180-day readmission rates, and EMS encounters) in the IG compared to the CG is not always reflected in the reduction of the proportion of patients having the event. This implies that the Stepped-Care approach is mainly effective on patients with multiple events. Figure 2 illustrates this finding for EMS encounters and 90-day readmissions by comparing the patients in the CG and IG that have single vs. multiple events. Figure 2a shows 29 (17%) vs. 20 (13%) patients having 53 vs. 25 EMS encounters in the CG vs. IG respectively during the 180-day intervention period. The number of patients having single EMS encounters were comparable in both groups—20 (11.6%) vs. 16 (10.0%) in the IG vs.Welo CG. However, there was 50% decrease in the number of patients with multiple EMS encounters—9 (5.2%) vs. 4 (2.5%) patients in the CG vs. IG. Also, the corresponding EMS encounters were fewer (9 vs. 33) in the IG vs. CG. Unlike the CG, where we had 4 patients with 4, 5, 6, and 7 EMS encounters respectively, no patients in the IG had more than 3 EMS encounters during the 180-day intervention period. Thus, the EMS encounters of heavy utilizers were substantially reduced. Figure 2b shows similar results for 90-day readmissions.

Our study population predominantly consisted of older patients with multiple chronic conditions similar to several previously published studies^17–19^. Therefore, support for multiple chronic conditions is a high priority in care management of older patients and the tailored interventions of the Stepped-Care approach used in this study could therefore be an effective strategy. Several studies in the literature have evaluated the effect of personalized interventions for chronic conditions including heart failure, depression, and risk of falling in seniors^20–23^. Results showed significant reductions in healthcare utilization similar to findings in this study. Complementing such personalized programs with predictive algorithms to target the interventions to high-risk patients may lead to more efficient use of healthcare resources.

Recent studies have reported the development and validation of predictive models that can be used to systematically identify individuals at high risk of eleven common cancers, cardiovascular disease, knee osteoarthritis and high inpatient utilization^24–28^. Such risk assessment models have great potential to inform treatment decisions and improve the quality of patients’ care. While PERS is traditionally known as a fall alert system, our prior analysis^14^ showed that it was used to request help for acute or chronic condition-related symptoms in addition to fall-related events, and significantly more symptom-related events resulted in ED transport compared with fall-related events. Further, the evidence from this study suggests that the CareSage predictive algorithm presents a unique opportunity to efficiently allocate limited healthcare resources to patients that need them the most and thereby reduce excessive healthcare utilization. Therefore, PERS as a remote patient monitoring technology can complement population health management programs by not only facilitating rapid medical response but also help in the early detection of patients at risk of high healthcare utilization.

Many HCOs develop population health management programs targeting T segment of patients who contribute to a sizeable portion of healthcare costs^11,12^. Although these programs have demonstrated improvement in clinical outcomes, they have not always yielded the necessary cost savings^29,30^. Targeting only high-cost patients may serve well under the fee-for-service model but prevents health systems from targeting more system-wide strategies that can improve outcomes in patients beyond the top segment and reduce the overall healthcare spending among their patient populations.

This study has some limitations. First, as shown in Table 1, the study population is mostly older, primarily female, white, patients who are living alone and are highly educated, thus limiting the generalizability of the study. Second, our analyses exclude clinical encounters not captured in the medical records or in the claims reported in the MGB. Furthermore, study patients with ED transport may not have ended up in the ED of one of the hospitals of the MGB network, but in a different hospital system, with no records of such ED encounters in the MGB EHR data that we used. To mitigate these limitations, we included questions related to the healthcare utilization in the close out survey. There were no substantial differences between the self-reported answers and the encounters recorded in the EHR and PERS data. However, all these limitations may have affected the correlations between outcomes like ED encounters in the IG and CG.

In conclusion, although many healthcare organizations target their most expensive patients to drive value-based care through improved outcomes, this randomized controlled trial highlights the potential to reduce healthcare utilization (90-, 180-day readmissions, and EMS encounters) in older patients in the middle segment using actionable predictive analytics combined with tailored interventions. The Stepped-Care approach may also help healthcare organizations manage their population health in low-cost settings such as home or community settings and facilitate the delivery of value-based care by improving health outcomes.

## Methods

### Design

This paper focuses on healthcare utilization as measured by the outcomes described below. Healthcare cost results associated with these outcomes will be presented in a separate manuscript^31^ because the cost data were not available until after the fiscal year 2019 was finalized, i.e. there was a delay of 8 months compared to the clinical outcomes.

The methods used in this study are summarized according to CONSORT (Consolidated Standards of Reporting Trials) recommendations^32^. A full description of the study protocol is available in^33^.

The study was implemented as a two-arm randomized controlled trial. All enrolled patients were randomized by the study coordinator via a computerized random-number generator to either the intervention group (IG) or the control group (CG). Treatment allocation was concealed in an opaque envelope opened after the informed consent procedures, so patients and the enrolling study staff were blinded to allocation until then.

The study period was 9-months divided into a 3-month observation period followed by a 6-month intervention period. The observation period was necessary for PERS data collection to make accurate predictions by the CareSage predictive model.

The study was approved by the Mass General Brigham Human Research Committee, the Institutional Review Board for MGB and registered as NCT03126565 at ClinicalTrials.gov, first posted on April 24th, 2017^34^.

### Participants

Study participants were identified among MGB patients receiving home care from PHH to manage their chronic conditions. Eligible patients were 65 years or older and English speaking. Further, PHH patients were identified as M segment patients if their total healthcare costs were within the middle (6th to 50th percentile) segment of the cost acuity pyramid for the fiscal year prior to their enrollment (2016 for patients enrolled in 2017; 2017 for patients enrolled in 2018). The M segment cutoffs for 2016 and 2017 were projected based on 2011–2015 data from the analysis that was the precursor to this prospective study^6^. Excluded from the study were patients with implanted devices (as a precautionary measure against possible interference with PERS), or suffering from dementia, Alzheimer’s or other psychiatric illness (anxiety disorder or psychosis). Patients with inpatient admissions resulting in total days hospitalized greater than 30 (i.e., 1/6 of the intervention period), or discharged into long-term-care facilities including Skilled Nursing Facilities, were excluded from the study. Finally, patients missing identifiers necessary to map the patient’s EHR and PERS data, thus preventing predictive model risk score calculation, were excluded as well, since the junction in the Stepped-Care approach at which the study nurse would contact a high-risk IG patient was precluded. Exclusions were applied regardless of group assignment. All patients provided informed written consent to take part in the study.

### Intervention

All patients received a PERS at home, connecting them to a response center any time they needed help during the study period. In addition, all participants were instructed to directly push the PERS help button or call 911 for connection to EMS if they experienced worsening of symptoms or required immediate attention.

**Fig. 3:**
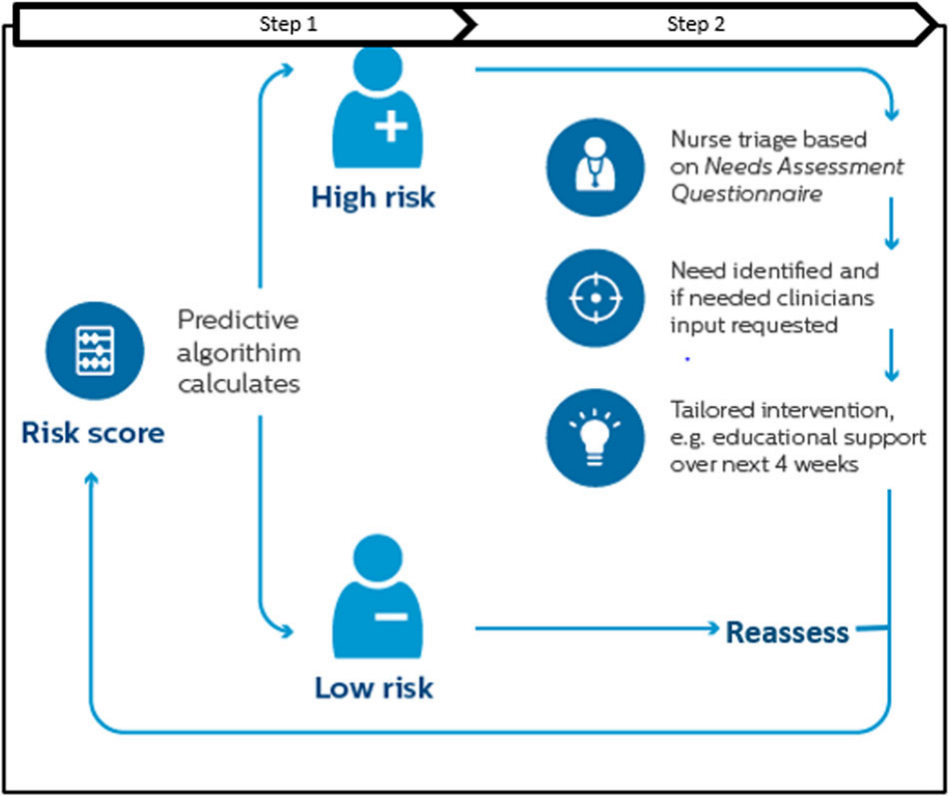
Intervention design—Stepped-Care approach. Figure 3 shows the (step one) predictive model ingesting data to calculate and generate risk scores, followed by (step two) nurse triage for patients flagged as high risk by the model. The figure also demonstrates how patients are regularly reassessed by the model.

During the 3-month observation period, all patients from the CG and the IG continued to receive care-as-usual from their care providers and no study interventions were administered. While the CG continued to receive care-as-usual during the next 6-month intervention period, the IG received care according to the Stepped-Care approach depicted in Fig. 3. As a first step, each IG patient’s risk of ED transport in the upcoming 30-days was assessed daily by the CareSage predictive model, starting on the first day of their intervention period. The CareSage predictive model includes three categories of predictors: (1) PERS utilization predictors such as the frequency and recency of various types of incidents at home (e.g., falls, respiratory issues, and chest pain) and their outcomes (e.g., responder assistance, EMS assistance, EMS transport to ED), (2) self-reported medical conditions provided at PERS enrollment (e.g., high blood pressure, diabetes, congestive heart failure), and (3) other predictors such as age, gender, and time on the PERS service. Machine learning was used to derive the predictive model and its Area under the Receiver Operator Characteristic Curve (AUC) was 78%. As a part of the validation, we compared the model’s predictions with clinical outcomes derived from the MGB electronic health record. One of the findings was that patients who were predicted to be high-risk had nearly four times higher rates of ED encounters than the low-risk patients^8^.

Patients not flagged as high-risk continued to receive care-as-usual. The study nurse viewed the CareSage dashboard to initiate an intervention tailored to any patient flagged as high-risk, as the second step of the Stepped-Care approach. The tailored intervention always started with a needs assessment questionnaire with the patient via telephone. The assessment included general health questions as well as assessments of respiratory symptoms, physical activity, activity of daily living, pain and bladder control^33^. Based on the patient’s needs and clinician’s input (if needed), a care plan was assigned including tailored feedback provided during the needs assessment and later follow-up as needed. Because this was a clinically diverse patient cohort, no single care plan was designated as an intervention prior to the study. Instead, a variety of care plan components—such as follow-up reassessments, patient education over a four-week period, home visits or outpatient visits to primary care physicians (PCP), or telemonitoring—were tailored to the patients by the study nurse based on individual patient needs. Thus, the study aims to evaluate the combined effect of both steps of the Stepped-Care approach on healthcare utilization, rather than a predefined care plan.

### Outcome measurements

To assess the healthcare utilization, the ED encounter rate in the IG and CG was selected as the primary outcome of this study. Secondary outcomes assessing healthcare utilization were EMS encounters, ED transport (upstream, occurring prior to the ED encounter), and hospital utilization (downstream, occurring post ED encounter). Hospital utilization was measured by hospital inpatient admissions, and 30-, 90-, and 180-day hospital readmission rates.

**Fig. 4:**
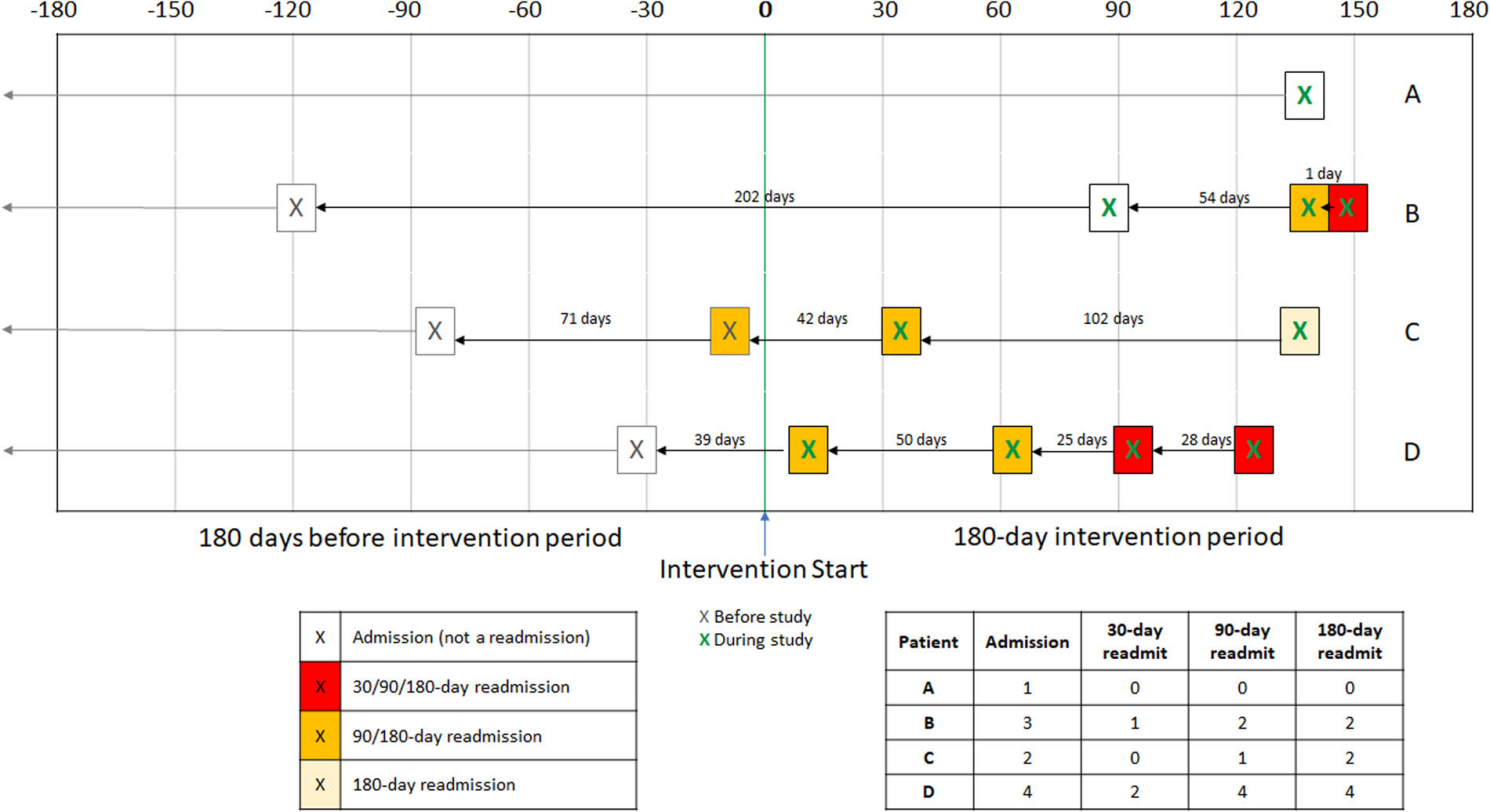
Visualizing admissions and readmissions. Figure 4 illustrates four patients’ examples on how admissions, 30-, 90-, and 180-day readmissions were counted in this study.

All outcomes were counted if they occurred between days 1–180 of the intervention period. Hospital admissions were defined as inpatient encounters which resulted in a patient being admitted to the hospital for any reason. After a patient’s hospital discharge, there is a risk of readmission. Such an event occurring within 30, 90, or 180 days of a prior discharge is considered a 30-, 90-, or 180-day readmission, respectively. Therefore, the type of readmission (30-, 90-, 180-day) is not relative to the start date of the intervention period, rather, it is relative to the prior (index) discharge date, which may have occurred before or during the intervention period. Regardless of when the index admission occurred, the readmission was counted so long as it occurred during the intervention period. See Fig. 4 for an illustration.

### Data collections

Patient baseline characteristics and patients’ needs were collected using enrollment and needs assessment questionnaires developed by the study investigators^33^. These data were maintained in and extracted from a REDCap database, which is a secure Web application for building and managing web-based surveys and databases.

All data pertaining to healthcare utilization were extracted from the MGB Enterprise Data Warehouse (EDW), which is a repository of clinical, operational, and hospital cost data of patients receiving care across MGB. These data are a source for analytics and reporting used by entities within the MGB and therefore considered reliable. PERS utilization data were collected from the Lifeline PERS database, which constitutes data on EMS encounters (emergency care provided at home), and ED transports (EMS transport to an ED). These data are regularly audited and used by response agents to help patients at home and are therefore considered reliable.

All patients’ data were de-identified before analyses. The data collected were aggregated and analyzed after the last patient closed out of the study.

### Statistical analysis

The sample size for the primary outcome was derived based on power analysis with the following parameters: two-armed randomized controlled design with 1:1 allocation ratio and a power of 0.80 at 2-tail significance level of α=0.05 with an intervention effect size of 0.35. This power calculation estimated a sample size of 160 patients per arm, which was adjusted for a lost to follow-up of 15%, leading to study total sample size of 370. The choice of intervention effect size of 0.35 was based on hypothesis that the Stepped-Care approach will be effective in reducing potentially avoidable hospitalizations with an ED admission source. Our previous retrospective analysis of similar population showed that these avoidable admissions were close to 35%^14^.

The intention-to-treat and per-protocol approaches were used for analyzing data^13^. Per-protocol analysis is a comparison of control and intervention groups that includes only those patients who completed the intervention originally allocated. Intention-to-treat analysis is a comparison of the control and intervention groups that includes all patients as originally allocated after randomization. This means healthcare events of patients who died, have dropped, were withdrawn, or lost to follow-up were included in the intention-to-treat analysis for the period they participated in the study.

Data were extracted retrospectively from REDCap, EDW and PERS databases, i.e., after the final study close-out date. All data analysis was performed using the statistical software R, version 3.6.1^35^.

Descriptive statistics on patients’ baseline characteristics were summarized by study group as well as total study population. They were derived as means and standard deviations (SDs), or frequencies and percentages. Comparisons of normally/non-normally distributed continuous variables by groups were conducted using two-sided Student t-tests/Mann-Whitney U tests, respectively. For categorical variables, Pearson Chi-square tests were used to examine the association between the groups.

All primary and secondary outcomes—ED encounters, hospital (re)admissions, EMS encounters, ED transports—were event-count variables. They were modeled using Poisson regression, which is a generalized linear model form of regression analysis used to model count data. Models were controlled for baseline characteristics which differed between groups if needed.

Due to multiple hypothesis testing, we applied Benjamini & Hochberg (BH) correction to control the false discovery rate, i.e., the expected proportion of false discoveries amongst the rejected hypotheses. This correction resulted into adjusted thresholds for statistical significance *α*_adj_ = α*i/m, where *α* = 0.05, *m* is the number of hypothesis tested, and *i* = 1, …,*m* is the position or the regression model *p*-value in an ordered list from smallest to largest. Then, only *p* < *α*_adj_ were called statistically significant.

### Reporting summary

Further information on research design is available in the Nature Research Reporting Summary linked to this article.

## Supplementary information

Reporting Summary

## Data Availability

The data that support the findings of this study are available from Mass General Brigham but restrictions apply to the availability of these data, which were used under IRB approval, and therefore we are not able to make this data publicly available. De-identified data are, however, available from the authors upon reasonable request and with permission from the Mass General Brigham Human Research Committee.

## Code availability

The code that supports the findings of this study is based on standard functions and packages available in R, which is an open-source language and environment for statistical computing. No proprietary code or/and mathematical algorithms are used.
